# First Comparative Analysis of the Community Structures and Carbon Metabolic Pathways of the Bacteria Associated with *Alvinocaris longirostris* in a Hydrothermal Vent of Okinawa Trough

**DOI:** 10.1371/journal.pone.0154359

**Published:** 2016-04-25

**Authors:** Qing-lei Sun, Zhi-gang Zeng, Shuai Chen, Li Sun

**Affiliations:** 1 Key Laboratory of Experimental Marine Biology, Institute of Oceanology, Chinese Academy of Sciences, Qingdao, China; 2 Key Laboratory of Marine Geology and Environment, Institute of Oceanology, Chinese Academy of Sciences, Qingdao, China; 3 Laboratory for Marine Biology and Biotechnology, Qingdao National Laboratory for Marine Science and Technology, Qingdao, China; 4 University of Chinese Academy of Sciences, Beijing, China; U.S. Geological Survey, UNITED STATES

## Abstract

*Alvinocaris longirostris* is a species of shrimp existing in the hydrothermal fields of Okinawa Trough. To date the structure and function of the microbial community associated with *A*. *longirostris* are essentially unknown. In this study, by employment of the techniques of high through-put sequencing and clone library construction and analysis, we compared for the first time the community structures and metabolic profiles of microbes associated with the gill and gut of *A*. *longirostris* in a hydrothermal field of Okinawa Trough. Fourteen phyla were detected in the gill and gut communities, of which 11 phyla were shared by both tissues. *Proteobacteria* made up a substantial proportion in both tissues, while *Firmicutes* was abundant only in gut. Although gill and gut communities were similar in bacterial diversities, the bacterial community structures in these two tissues were significantly different. Further, we discovered for the first time the existence in the gill and gut communities of *A*. *longirostris* the genes (*cbbM* and *aclB*) encoding the key enzymes of Calvin-Benson-Bassham (CBB) cycle and the reductive tricarboxylic acid (rTCA) cycle, and that both *cbbM* and *aclB* were significantly more abundant in gill than in gut. Taken together, these results provide the first evidence that at least two carbon fixation pathways are present in both the gill and the gut communities of *A*. *longirostris*, and that the communities in different tissues likely differ in autotrophic productivity.

## Introduction

Since the first discovery of hydrothermal vents in 1977, the unique and diverse biological communities associated with the seafloor hydrothermal systems have drawn a great deal of attention [1–5]. Microorganisms in deep-sea hydrothermal environments possess various metabolic capacities such as carbon fixation, sulfur oxidation, and nitrification and denitrification; as a result, they widely participate in the cycling of carbon, nitrogen, and sulfur in the ecosystems [6–8]. A number of studies on microbial communities in deep-sea hydrothermal systems, including chimney structures, sediments, and fluids, have been reported [9–12]. Many species of invertebrates dwelling in hydrothermal vents are known to host microbes in the form of epibionts or endosymbionts. Epibionts adhere to the surface of specialized tissues of the host, such as the dorsal setae of the polychaete *Alvinella pompejana*, the gill chambers of the shrimp *Rimicaris exoculata*, and the setae of the galatheid crabs *Shinkaia crosnie*ri [13–15]. Endosymbionts inhabit inside the cells of host [16–18].

The commensal microbial communities associated with megafauna in hydrothermal vents are believed to have chemolithoautotrophic abilities which enable the microbes to provide continuous nutrition to their hosts [15, 19]. The autotrophic productivity of the symbiotic microorganisms in *R*. *exoculata*, *S*. *crosnieri*, and *A*. *pompejana* was supported by detection of genes encoding key enzymes of carbon fixation and by *in vivo* or *in vitro* experiments that demonstrate carbon assimilation [15, 19, 20–22]. In addition, considering that toxic H_2_S and heavy metals are rich in hydrothermal environments, the symbionts might also play a detoxification role that facilitates the survival of the host animals [23, 24].

The shrimp *Alvinocaris longirostris* belongs to *Alvinocarididae* family and is abundant in the hydrothermal vent fields of Okinawa Trough [25, 26]. To date, only one study on *A*. *longirostris*-associated microbial community has been documented, in which the dominant gill bacteria of *A*. *longirostris* was analyzed by restriction fragment length polymorphism analysis of a 16S rDNA clone library [26]. It was found that among the 48 clones analyzed, approximately 70% were *Sulfurovum* sp. AL-1. However, given its limited detecting ability, this low-profiling tool is usually unable to discover rare species in complicated environmental samples [27]. As a result, diversity of communities associated with *A*. *longirostris* remains largely unknown. In addition, there has been no molecular evidence on the chemoautotrophic capacities of the microbial communities associated with *A*. *longirostris*.

In the current work, we aimed to investigate the community diversity and autotrophic potential of microbes associated with the gill and gut of *A*. *longirostris* from a hydrothermal vent of Okinawa Trough. For this purpose, we employed Illumina sequencing platform, a high through-put sequencing technique, to examine the microbial communities associated with the gill and gut of *A*. *longirostris*. In addition, by clone library analysis and quantitative PCR analysis, we analyzed the existence and abundance of the genes encoding the key enzymes of two major carbon metabolic pathways.

## Materials and Methods

### Ethics statement

The sampling location was not privately owned or protected in any way, and no specific permits were required for the study of sampling field or for sample collection. The object study field did not involve any endangered or protected species. Live animal researches were performed in accordance with the "Regulations for the Administration of Affairs Concerning Experimental Animals" promulgated by Shandong Province. The protocol was approved by the Ethics Committee of Institute of Oceanology, Chinese Academy of Sciences.

### Sample collection

*A*. *longirostris* specimens were sampled at the Iheya Ridge hydrothermal field in Okinawa Trough (126.97°E, 27.55°N, 1243 m deep) during the “Okinawa Trough hydrothermal fields Cruise” in April, 2014. The shrimp were collected using a slurp gun equipped on the remotely operated vehicle (*R/V*) *Faxian*. Once aboard, the specimens were immediately thoroughly washed with sterile seawater and frozen at −80°C. In the laboratory, five shrimps were used for the study and treated as follows: each animal was dissected separately to take out the gut and gill; the gill and gut from shrimp 1 were named Gill 1 and Gut 1, respectively, and the other samples were named in the same manner. The resulting 10 tissue samples (two from each animal) were then used to construct 10 individual libraries. For this purpose, the tissues were weighed and used to extract DNA using a FastDNA SPIN Kit (MP Biomedicals, Santa Ana, USA) according to the manufacturer’s instructions. The quality and size of the extracted genomic DNA were assessed by 1.0% agarose gel electrophoresis.

### Library preparation and sequencing

The concentration of the DNA was determined with NanoDrop ND-2000 (Thermo Scientific, Wilmington, USA). The V3-V4 regions of the 16S rRNA gene were amplified from each sample using universal prokaryotic primers with barcodes as reported previously [28]. The universal primers matched approximately 98.0% of Bacteria and 94.6% of Archaea rRNA gene sequences in the Ribosomal Database Project database [29]. Sequencing libraries were generated using NEB Next® Ultra™ DNA Library Prep Kit for Illumina (New England Biolabs, USA). The library quality was assessed with Qubit@2.0 Fluorometer (Thermo Scientific, USA) and Agilent Bioanalyzer 2100 system. The library was sequenced on an Illumina MiSeq platform at Novogene (Beijing, China), and 300 bp paired-end reads were generated.

### Data analysis

Paired-end reads were assigned to each sample according to the unique barcodes. The raw reads were merged to raw sequence tags using FLASH [30] and filtered using an average quality value of 20 (Q20). Effective sequence tags were obtained by further splicing, filtering, and removing chimeric sequences [31]. Effective sequence tag analysis was performed using the UPARSE-OTU and UPARSE-OTUref algorithms [32]. Sequence tags with ≥ 97% identity were assigned to the same OTU (operational taxonomic unit). A representative sequence tag was picked for each OTU, and RDP classifier was used to annotate taxonomic information for each representative sequence tag with a bootstrap cutoff of 50% [33]. The software program mothur was used to analyze alpha diversity (Shannon index and Chao1) [34–36]. Rarefaction curves were generated based on the number of OTUs using QIIME software package (Version 1.7.0) [37]. In a second analysis, the OTUs were analyzed using the BLAST program at the National Center for Biotechnology Information (NCBI) (http://www.ncbi.nlm.nih.gov) [38]. Neighbor-joining trees were constructed with Kimura two-parameter distance with bootstrap analysis of 1000 data sets using MEGA 5.0 software [39].

In beta diversity analysis, two dimensional Principal Coordinates Analysis (PCoA) based on weighted UniFrac distances were performed to visualize broad trends of similarities and differences that related all samples using the QIIME software package. The discrimination of microbial community structures of different groups was tested with analysis of similarities (ANOSIM) using PRIMER 6 [40].

### Cloning, sequencing, and phylogenetic analysis of the genes encoding ATP-dependent citrate lyase (*aclB*) and the large subunit of RubisCO form II (*cbbM*)

DNA was extracted from the gill and gut samples of five individual shrimp; Equal amounts of DNA preparations from the same tissue samples were mixed and used as PCR templates. The *aclB* and *cbbM* genes were amplified using the primer sets 892F/1204R and cbbMF/cbbMR, respectively [19, 41]. The PCR conditions were as described previously [19, 41]. The amplified PCR products were purified using OMEGA Gel Extraction Kit (OMEGA bio-tek, Georgia, USA) and cloned into pEASY-T (TransGen Biotech, Beijing, China). Fifty colonies were picked randomly from each group and were checked by direct PCR with the vector primers M13F and M13R. Positive colonies with the correct insert were sequenced in BGI, Beijing, China. The amino acid sequences of the genes were aligned using Mega 5.0 [39], and sequences displaying more than 95% identity were clustered within the same OAU (operational AclB unit) or OCU (operational CbbM unit) using mothur software [34]. Good’s coverage was calculated based on 95% identity as described previously [42, 43]. Phylogenetic analysis was performed as above.

### Quantification of *aclB* and *cbbM* genes

The abundance of *aclB* and *cbbM* genes was determined by a quantitative real-time PCR method using the same primers and conditions for cloning these genes described above. The  PCR was carried out in an Eppendorf Mastercycler (Eppendorf, Hamburg, Germany) using SYBR ExScript RTqPCR Kit (Takara, Dalian, China) as described previously [44]. Melting curve analysis was performed at the end of PCR to confirm that only one PCR product was amplified and detected.

### Nucleotide sequence submission

The high through-put sequencing data had been submitted to the NCBI Sequence Read Archive database under the accession numbers SRX1352943 (gill) and SRX1352973 (gut). The nonredundant sequences of *aclB* and *cbbM* reported in this study have been deposited in GenBank under accession numbers KT932714-KT932740.

### Statistical analysis

Statistical analysis was carried out with SPSS 17.0 software (SPSS Inc., Chicago, IL, USA). Data were analyzed with analysis of variance (ANOVA), and statistical significance was defined as *p* < 0.05.

## Results

### Quality filtering and counting of the sequencing data

Ten libraries were constructed from the gill and gut of *A*. *longirostris*. A total of 929061 raw reads were obtained from these libraries with Illumina Miseq sequencing technique. After merging and processing of the raw reads, 388169 effective sequence tags with an average length of >400 bp of the 16S rDNA spanning the variable regions V3 and V4 were recovered, of which 56.6% and 43.4% were from the gill and gut libraries, respectively (Table 1).

**Table 1:** Summary of the sequencing information.

	Gill-1	Gill-2	Gill-3	Gill-4	Gill-5	Gut-1	Gut-2	Gut-3	Gut-4	Gut-5
Effective sequence tags	53665	67328	43916	28452	26320	29545	35947	37448	31444	34104
Taxon tags	53491	67300	43916	28249	26320	29545	35947	37448	31444	34104
Unclassified tags	174	28	0	203	0	0	0	0	0	0
OTU	107	107	110	120	134	108	98	121	89	148
Shannon (H’)	2.26	1.88	2.32	2.47	2.84	3.11	2.07	2.93	1.28	1.84
Chao1	127.0	136.6	130.3	129.6	142.6	124.2	114.2	128.7	129.6	155.7

### Alpha and beta diversity analysis

**Fig 1:**
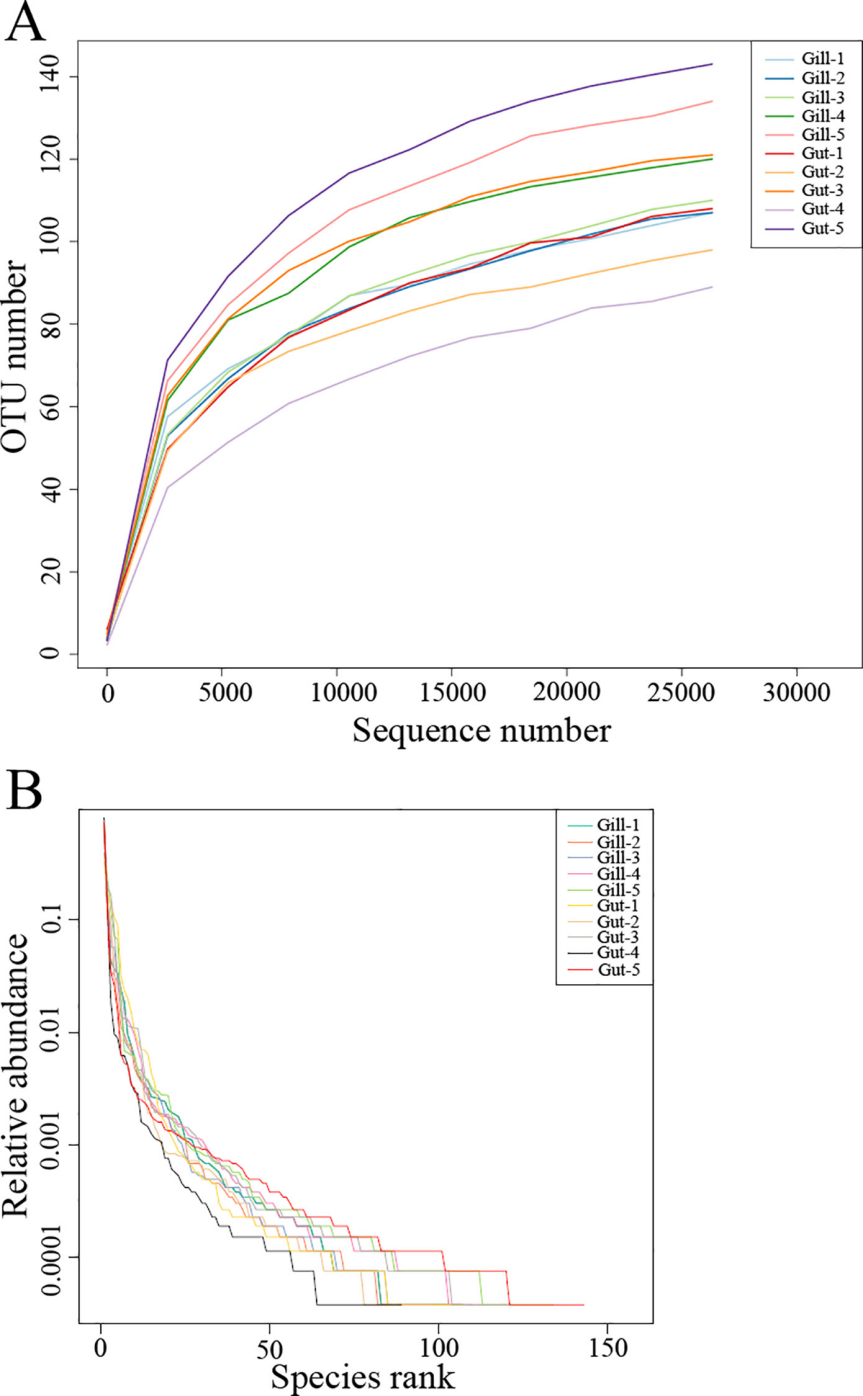
Diversity of microbial communities in each sample. Rarefaction curve (A) and rank-abundance curve (B) based on OTUs at a dissimilarity level of 3%.

**Fig 2:**
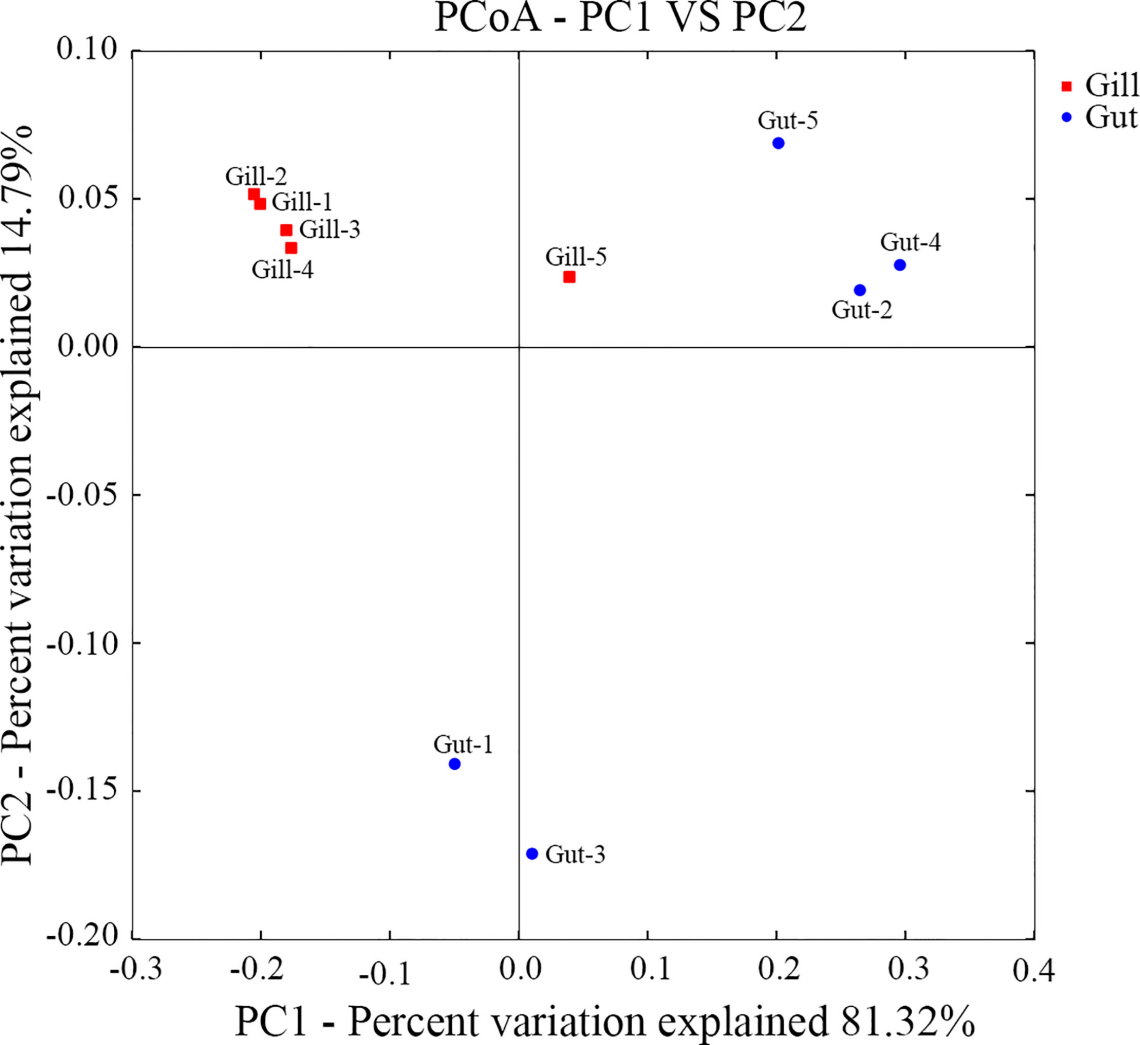
Principal Coordinate Analysis (PCoA) based on weighted UniFrac distance for all samples calculated with QIIME.

In alpha diversity analysis, the mean Shannon-Wiener diversity indices in gill and gut communities were 2.35 and 2.25, respectively, which were not significantly different (*p* = 0.781) (Table 1). Likewise, the mean Chao1 indices in gill and gut communities (133.2 and 130.5, respectively) showed no significant difference (*p* = 0.722) (Table 1). Rarefaction analysis based on OTUs at 3% dissimilarity indicated that all libraries represented well the microbial communities (Fig 1A). Rank-abundance curves showed that the majority of sequence tags were represented by a small amount of microorganisms, and all samples contained relatively low-abundance but highly diverse sequence tags (Fig 1B). In beta diversity analysis, it was observed that PCoA exhibited a clear separation, and that the gill samples (except Gill-5) were grouped to the left of the graph along PC1, which accounted for 81.32% of the total variations, whereas the gut samples (except Gut-1) were separated from the gill samples and grouped to the right of the graph (Fig 2). Further, ANOSIM analysis indicated that the microbial community structures in these two groups were significantly (*p* = 0.013) different.

### Microbial community analysis

**Fig 3:**
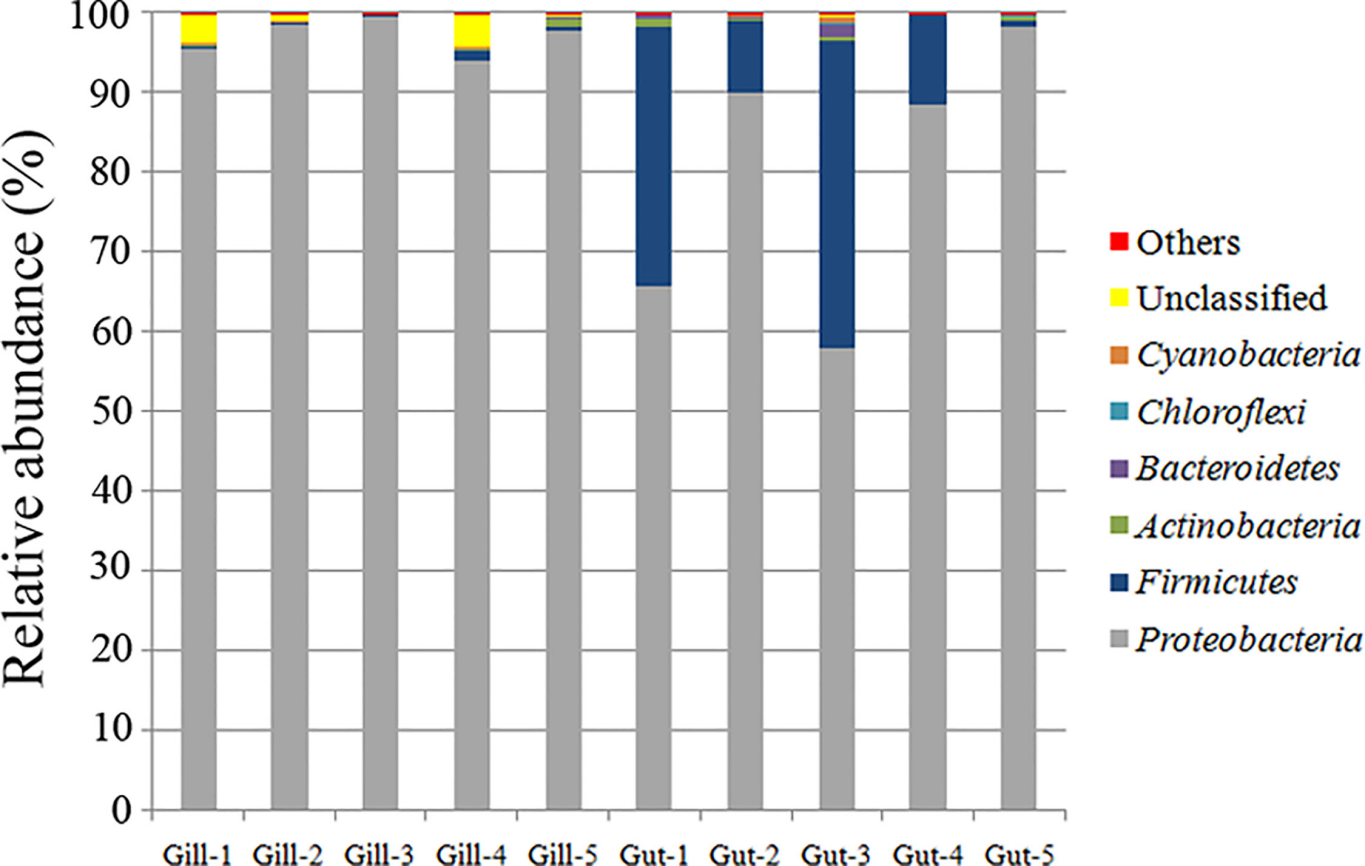
Diversity and distribution of microbial sequence tags in gill and gut libraries of *Alvinocaris longirostris*. The sequence tags were classified at the phylum level. Each color represents the percentage of the taxon in the total assemblage. Dominant groups (relative abundance ≥0.1%) are labeled, and minor groups are grouped as “Others”.

Nearly all sequence tags (99.9%) were assigned to bacterial domain, and the rest tags were unclassifiable. No archaeal sequence tags were detected in either sample. Most sequence tags were classified at the phylum (99.0%), class (98.9%), and family (89.5%) level. There were 14 phyla detected in gill and gut, among which 11 phyla were shared by both tissues, i.e. *Proteobacteria*, *Firmicutes*, *Actinobacteria*, *Bacteroidetes*, *Chloroflexi*, *Cyanobacteria*, TM7, *Verrucomicrobia*, OD1, *Tenericutes*, and *Thermi*. *Caldithrix* was only found in gut community, whereas *Spirochaetes* and *Planctomycetes* were only found in gill community. *Proteobacteria* was dominant in the two tissues, accounting for 97.1% and 80.1% of the sequence tags in gill and gut, respectively (Fig 3). *Firmicutes* was abundant only in gut (average 18.5%) (Fig 3). The other phyla were minor groups (< 1.0%) in both tissues.

**Fig 4:**
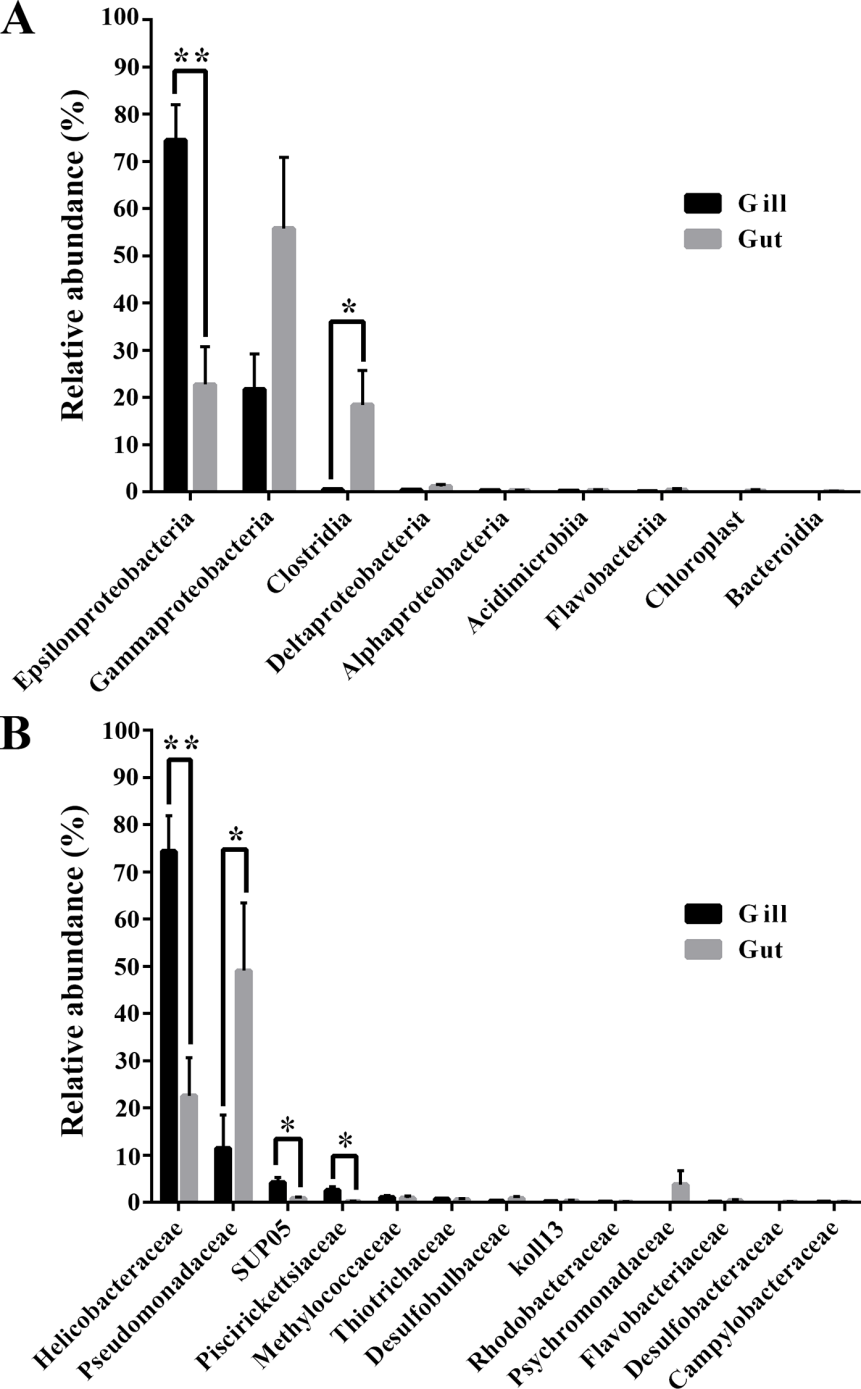
**Relative abundance of the main classes (A) and families (B) in the gill and gut libraries of *Alvinocaris longirostris* (relative abundance ≥ 0.1% at least in one tissue).** Error bars indicate standard errors. *p < 0.05, **p < 0.01.

Of the 20 classes identified, 17 were shared by gill and gut. *Epsilonproteobacteria* and *Gammaproteobacteria* of the phylum *Proteobacteria* were dominant classes in both tissues but varied in abundance, with *Epsilonproteobacteria* accounting for 74.5% of the sequence tags in gill, which was significantly (*p* = 0.002) higher than that (22.7%) in gut (Fig 4A). In contrast, *Gammaproteobacteria* in gut constituted 55.8% of the sequence tags, which was much higher but not significantly different (*p* = 0.077) from that (21.8%) in gill (Fig 4A). *Deltaproteobacteria*, *Alphaproteobacteria*, and *Betaproteobacteria*, which also belonged to the phylum *Proteobacteria*, were detected in both tissues as well but in low abundances (Fig 4A). *Clostridia* of the phylum *Firmicutes* was abundant only in gut, constituting 18.5% of the sequence tags, which was significantly (*p* = 0.039) higher than that (0.5%) in gill (Fig 4A). The other classes were less than 1.0% in both tissues.

Of the 50 families identified, 43 were shared by gill and gut. *Helicobacteraceae* dominated in gill, accounting for 74.4% of the sequence tags, which was significantly (*p* = 0.002) higher than that (22.6%) in gut (Fig 4B). *Pseudomonadaceae* constituted 49.1% and 11.5% of the sequence tags in gut and gill, respectively (Fig 4B). SUP05 (4.2%) and *Piscirickettsiaceae* (2.6%) were significantly more abundant in gill than in gut (Fig 4B). *Methylococcaceae* made up 1.1% and 1.0% of the sequence tags in gill and gut, respectively. The other families were less than 1.0% in abundance in both tissues.

### Phylogenetic analysis at low taxonomic level

**Fig 5:**
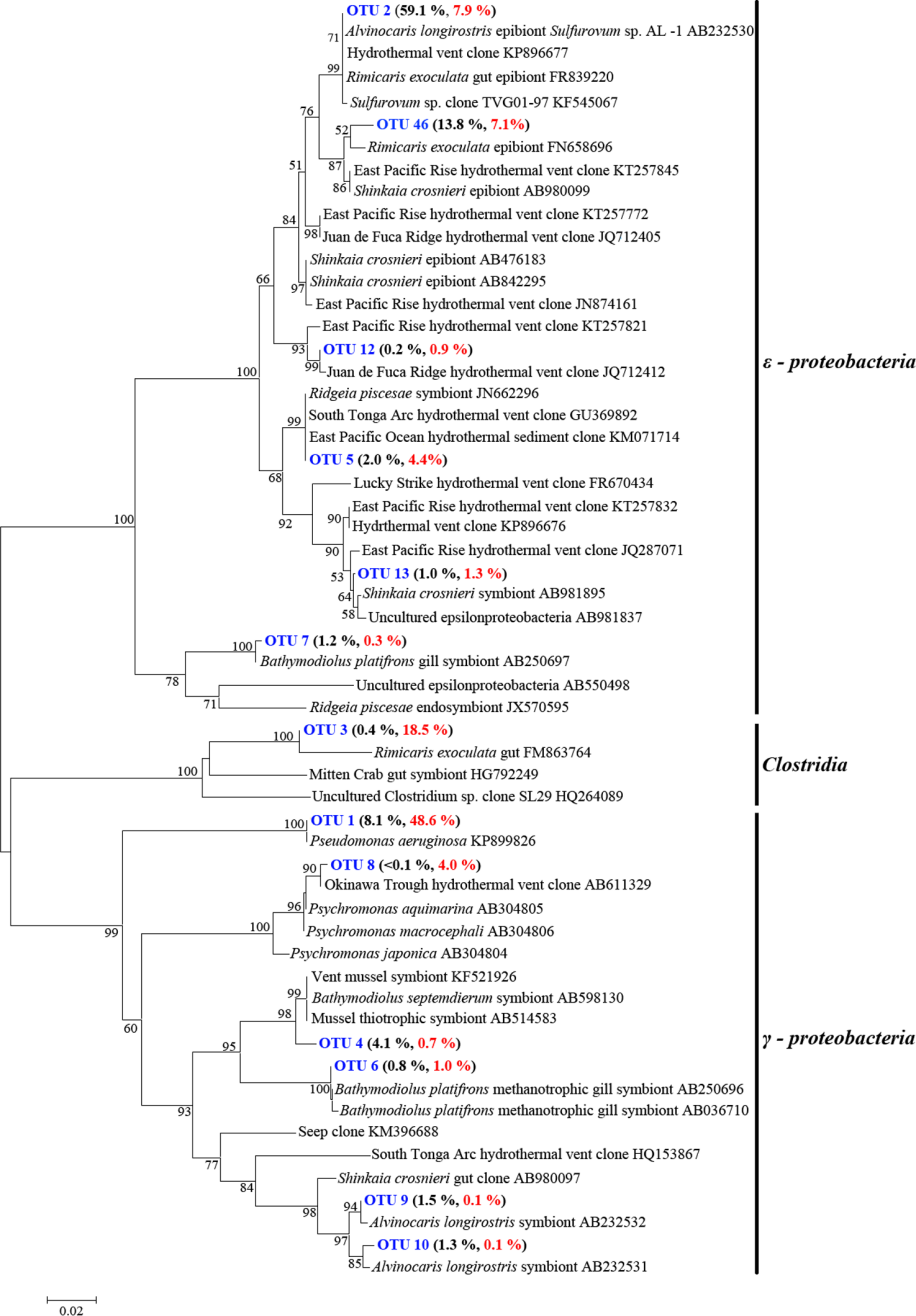
Phylogenetic positions of the top 10 OTUs of the gill and gut libraries of *Alvinocaris longirostris*. The tree was constructed using sequences longer than 400 bp. Neighbor-joining analysis with 1000 bootstrap replicates was used to infer the topology. Sequences obtained in this study are in blue. Black and red numbers in brackets represent relative abundances of the OTU in gill and gut, respectively. Scale bar represents 2% estimated sequence divergence.

Only 4.0% of the sequence tags were assigned to known genera. These sequences were distributed in 36 genera (S1 Table), including members of sulfur oxidizing or reducing bacteria, such as *Leucothrix*, *Sulfurimonas*, *Sulfuricurvum*, *Sulfurospirillum*, and *Desulfocapsa*, and a member of methylotropher, *Methylobacterium*. Based on 97% identity level, 192 OTUs and 186 OTUs assigned to gill and gut communities, respectively. Venn diagrams showed that 160 OTUs were shared by both tissues (S1 Fig). Phylogenetic analysis of the ten most abundant OTUs in gill and gut were carried out (Fig 5). These OTUs constituted 93.9% of the sequence tags of gill and gut, and most of which were unclassifiable at genus level. OTU2, constituting 59.1% and 7.9% sequence tags in gill and gut, respectively, was 100% identical to *Sulfurovum* sp. AL-1, an epibiont from the gills of *A*. *longirostris* [26]. OTU1, constituting 8.1% and 48.6% of the sequence tags in gill and gut, respectively, was related to *Pseudomonas aeruginosa* with a high identity (100%) and a high bootstrap value (100) (Fig 5). Most OTUs were highly related (identities 97% to 100%) to the clones from hydrothermal systems including sediments, fluids, and megafauna symbionts (Fig 5).

### Diversity and quantitation of carbon metabolic genes in gill and gut communities

**Fig 6:**
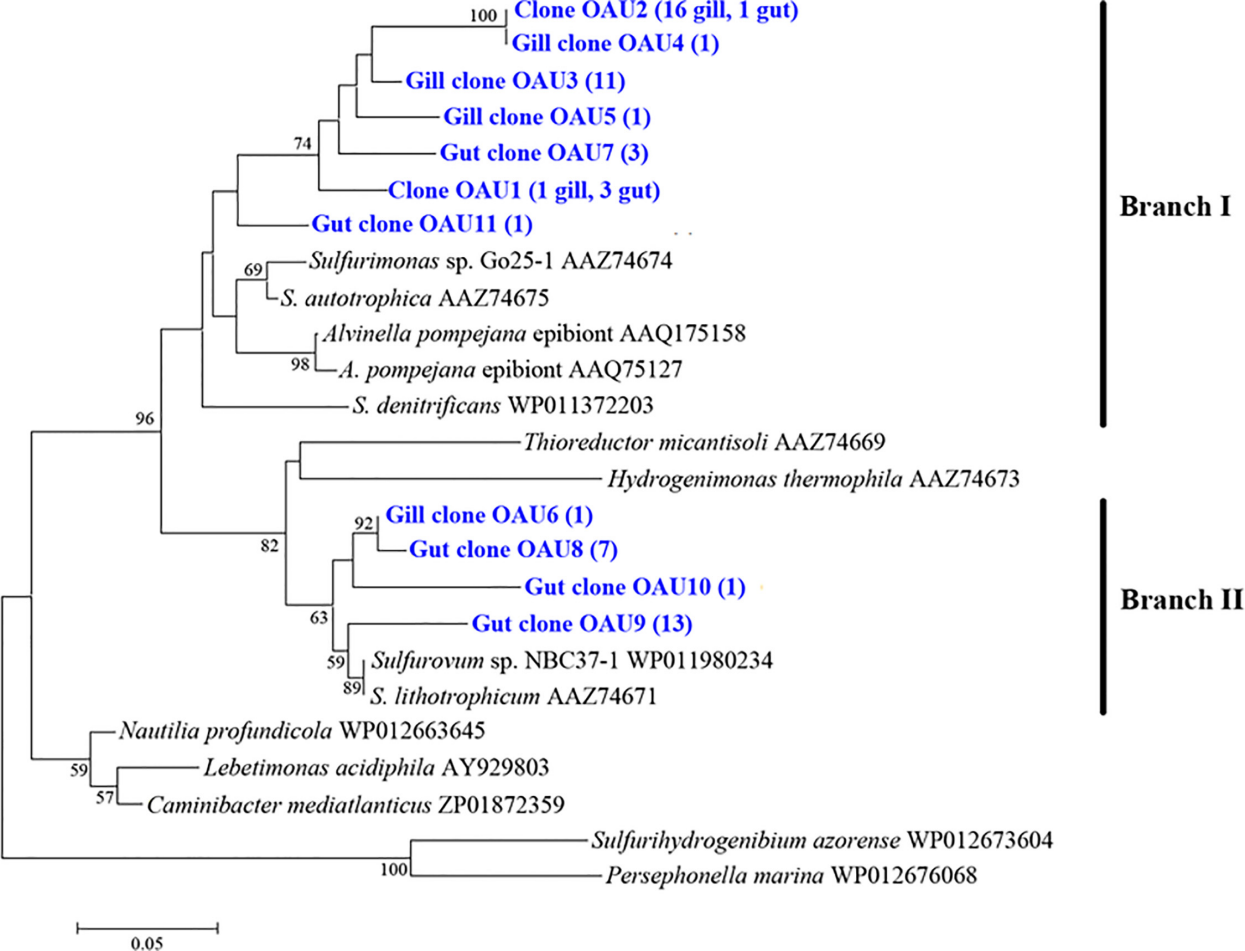
Phylogenetic tree based on AclB sequences. The tree was calculated with Neighbor-joining method. Bootstrap values are shown as percentages of 1000 bootstrap replicates. OAUs (operational AclB units) are shown in blue. Scale bar represents 5% estimated sequence divergence.

**Fig 7:**
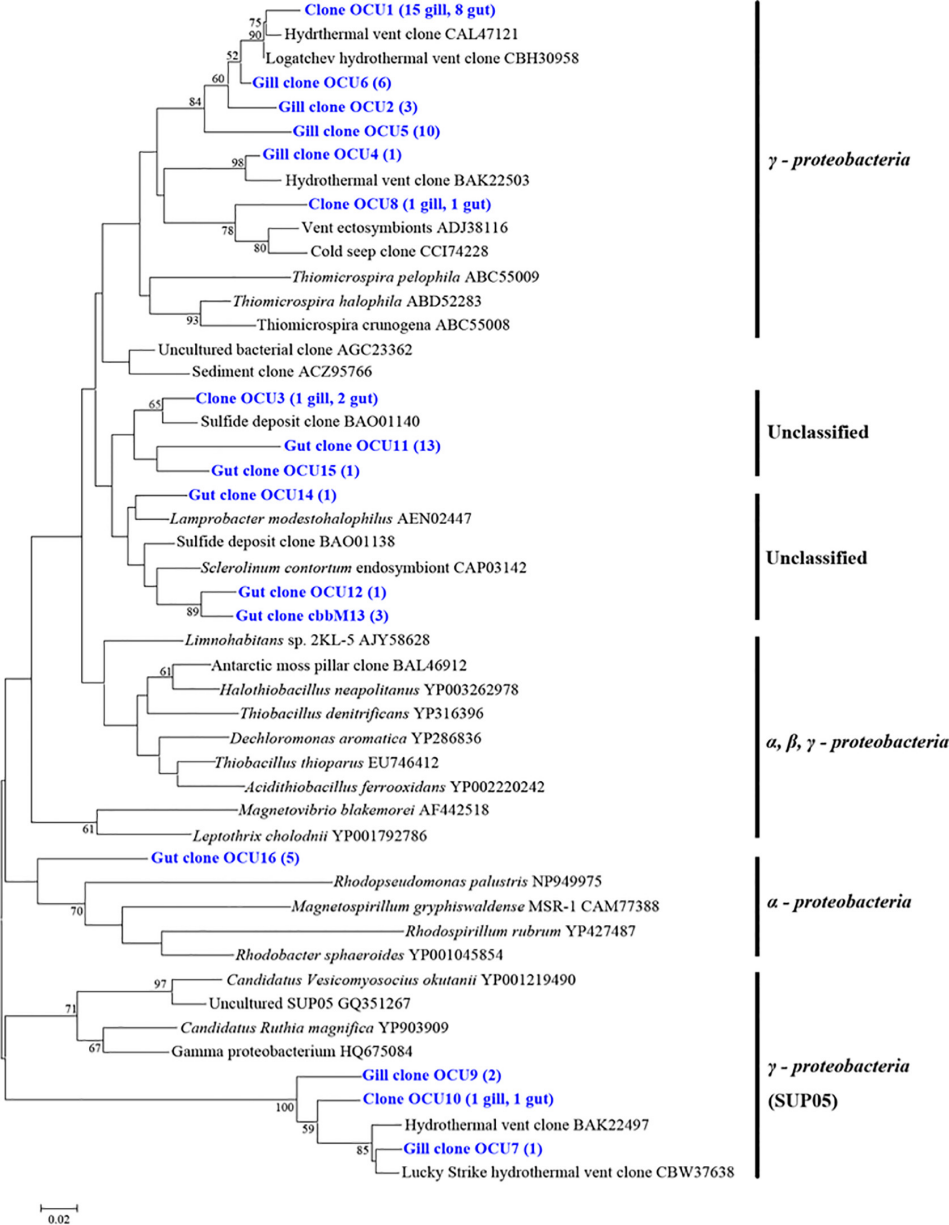
Phylogenetic tree based on CbbM sequences. The tree was calculated with Neighbor-joining method. Bootstrap values are shown as percentages of 1000 bootstrap replicates. OCUs (operational CbbM units) are shown in blue. Scale bar represents 2% estimated sequence divergence.

**Fig 8:**
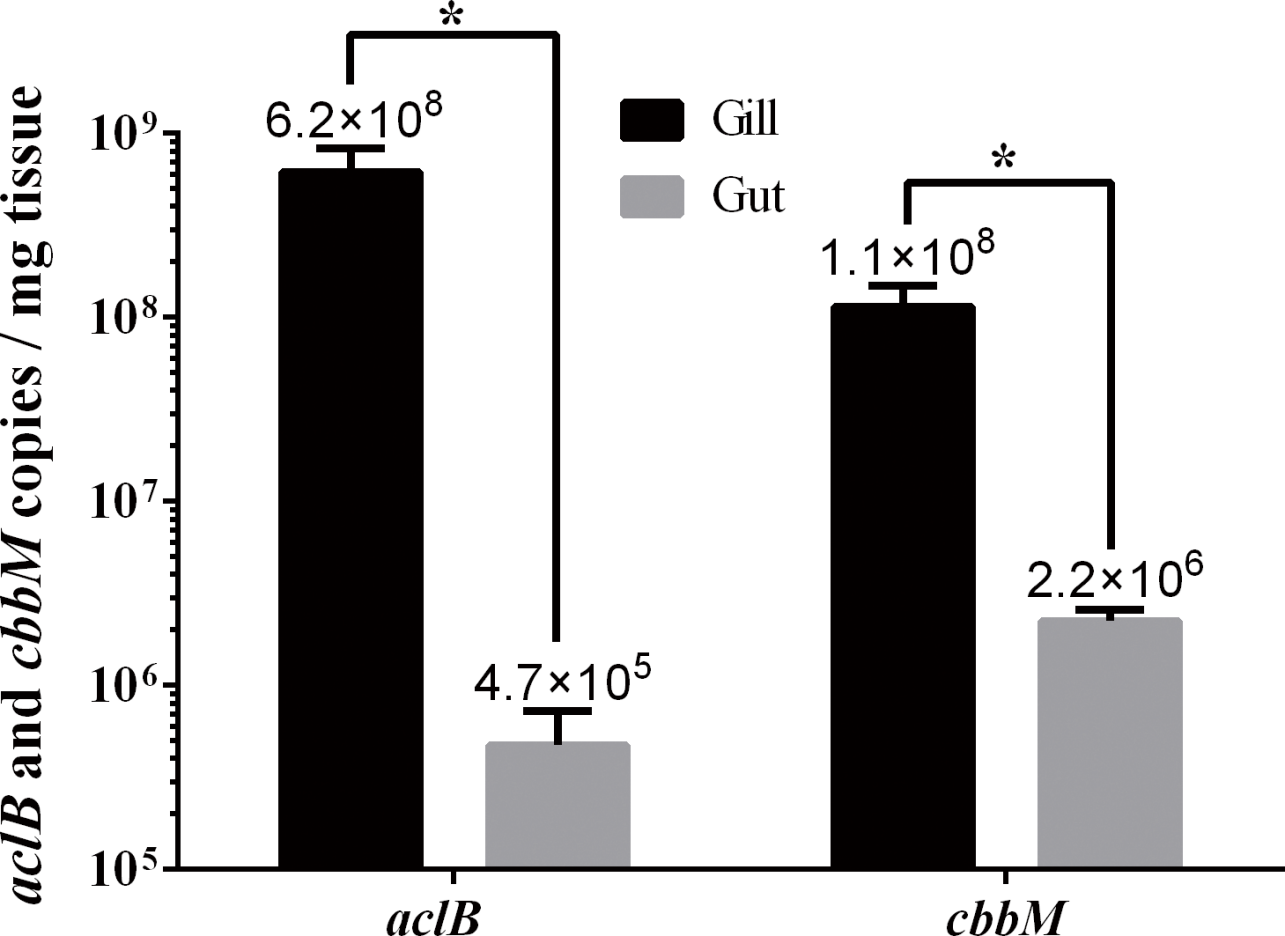
Abundance of *aclB* and *cbbM* genes in the gill and gut communities of *Alvinocaris longirostris*. Errors bars represent standard errors of five samples. *p < 0.05.

To assess the autotrophic potential and carbon fixation pathways of the microbial communities, clone libraries were constructed for the genes encoding the small subunit of ATP-dependent citrate lyase (*aclB*) and the large subunit of RubisCO form II (*cbbM*), which are the key enzymes of reductive tricarboxylic acid (rTCA) cycle and Calvin-Benson-Bassham (CBB) cycle, respectively. The Good’s coverage values of the *aclB* and *cbbM* libraries were 91.7% and 93.5%, respectively. A total of 60 *aclB* clones and 77 *cbbM* clones were sequenced. Based on the deduced amino acid sequences of these clones, 11 OAUs (operational AclB units) and 16 OCUs (operational CbbM units) were generated at 95% identity level. The AclB sequences were separated into two branches, both, however, belonging to a single clade composed of members of *Epsilonproteobacteria* (Fig 6). Branch I contained 7 OAUs representing 38 sequences (30 from gill, 8 from gut), which were related to *Sulfurimonas* genus; Branch II contained 4 OAUs representing 22 sequences (1 from gill, 21 from gut), which were related to *Sulfurovum* genus (Fig 6). For CbbM, 10 OCUs were distributed in two classes and six OCUs were unclassifiable (Fig 7). OCU7, OCU9, and OCU10 representing 5 clones (4 from gill, 1 from gut) formed a deep branch in *Gammaproteobacterial* SUP05 group; OCU1, OCU2, OCU4, OCU5, OCU6, and OCU8 representing 45 clones (36 from gill, 9 from gut) were also related to *Gammaproteobacteria* but separated distinctly from the branch of OCU7, OCU9, and OCU10; one gut clone, OCU16, was clustered into *Alphaproteobacteria* (Fig 7). Absolute quantitative RT-PCR analysis showed that the amounts of both *aclB* and *cbbM* in gill were significantly higher than that in gut (Fig 8).

## Discussion

### The gill community

In this study, we for the first time comparatively analyzed the microbial communities associated with the gill and gut of the hydrothermal vent shrimp *A*. *longirostris* using Illumina sequencing platform, a high through-put sequencing technique. Illumina sequencing platform is a powerful tool for studying microbial ecology in various environments [45]. Due to its in-depth sequencing capacity, Illumina can theoretically detect the diversity of an entire microbial community [27]. Further, Illumina MiSeq sequencer, the enhanced Illumina platform, can generate longer sequence reads (~600 bp) and thus provide more valuable information to assess microbial community structure [29]. In our study, we found that in the gill community, *Epsilon-* and *Gamma*-proteobacteria were the dominant groups of bacteria, while *Alpha*-, *Beta*-, and *Delta*-proteobacteria, *Clostridia*, and *Flavobacteria* were the minor groups. A similar community structure was detected in the gill-chamber of the shrimp *R*. *exoculata* from the hydrothermal field in Mid-Atlantic Ridge by a metagenomic approach [46]. In the low taxon level analysis, we detected many genera in the gill community of *A*. *longirostris*, including *Sulfurovum*, *Leucothrix*, *Sulfurimonas*, *Sulfurospirillum*, *Desulfocapsa*, and *Polaribacter*, some of which are known to possess detoxic abilities [23, 24]. The identification of *Sulfurovum* in our study was consistent with a previous report which showed that *Sulfurovum* was dominant in the gills of *A*. *longirostris* from a different site in Okinawa Trough [26]. These results suggest that the gill-associated bacteria may facilitate the survival of the host in the hostile hydrothermal environment.

To date, the original sources and the transmission processes of the microorganisms that form the gill community of hydrothermal vent shrimps remain unclear. It has been proposed that for epibionts that are also found in free-living form in the outside environment, horizontal transmission is possible [47]. In our study, most gill OTUs were closely related to sequences from hydrothermal sediments and fluids in Okinawa Trough as well as other vent sites in East Pacific Rise, Juan de Fuca Ridge, South Tonga Arc, and Mid-Atlantic Ridge (Lucky Strike vent field). These results support the theory of horizontal transmission. In addition, our study showed that the microbial diversity of the gill community was much lower than that in the sediments from the same vent site [28], and that although archaea are known to exist in the hydrothermal fields of Okinawa Trough and other hydrothermal vents [10, 11, 28], there were no archeal sequences detected in gill. These observations suggest that a filtering or selective process has likely occurred during horizontal transmission [48].

### The gut community

Before this study, there had been no documented research on the microbial community associated with the gut of *A*. *longirostris*. In this study, we found that most bacterial OTUs were shared by gill and gut. This observation, which is similar to that observed in the hydrothermal shrimp *R*. *exoculata* in Mid-Atlantic Ridge [48], suggests the possibility that the gut bacteria may be derived from the gill epibionts or from the outside environment. However, a marked difference also existed between gut and gill communities. It is possible that this difference is due to a filtering or selective process that operates during the formation of gut- as well as gill-associated microbiota, which results in tissue-specific OTUs. In line with this hypothesis, a recent study suggested an adhesion-as-selection model in the human gut, in which mucus glycans and immunoglobulin A were considered as candidate selective molecules that may help shaping the community structure [49]. ANOSIM analysis indicated a significant difference between the community structures of gill and gut, most obviously reflected in the observation that *Epsilonproteobacteria* was a dominant population in gill but not in gut, and that *Clostridia* was much more abundant in gut (18.5%) than in gill (0.5%). It is likely that the difference between gill and gut is caused by the different physico-chemical conditions in these tissues that exert a shaping and selection effect on the microbial community, resulting in the formation of distinct tissue-specific population structures.

### Carbon fixation

In our study, we detected *aclB* and *cbbM* genes in the gill and gut of *A*. *longirostris*, suggesting that the rTCA and CBB pathways of carbon fixation existed in the microbial communities of *A*. *longirostris*. A similar observation, i.e., co-occurrence of rTCA and CBB pathways, was also made in *Rimicaris exoculata* [20]. Phylogenetic analysis grouped some CbbM sequences within the *Gammaproteobacterial* SUP05 family, which is consistent with the fact that *Gammaproteobacterial* SUP05 was found in 16S rDNA libraries. These results suggest that members of SUP05 in the gill and gut communities possibly assimilate carbon through the CBB cycle. Likewise, phylogenetic analysis showed that all AclB sequences belonged to *Epsilonproteobacteria* and were clustered into the clades of *Sulfurimonas* and *Sulfurovum*, which was consistent with the detection of *Sulfurimonas* and *Sulfurovum* in 16S rDNA libraries. It is possible that members of *Sulfurimonas* and *Sulfurovum* in the gill and gut communities of *A*. *longirostris* fix carbon through the rTCA cycle. In addition, since SUP05, *Sulfurimonas* and *Sulfurovum* are in general sulfur oxidizers [50–53], their presence in the gill and gut of *A*. *longirostris* suggests a possibility that these bacteria may generate energy through oxidation of reduced sulfur compounds to drive carbon fixation. Quantitative PCR analysis revealed that for both *aclB* and *cbbM* genes, significantly more copies were found in gill than in gut, suggesting that the gill community has a higher autotrophic productivity and thus likely plays a more significant role as a nutritional source for the host.

## Conclusion

In this study, we demonstrate for the first time that different microbial community structures exist in the gill and gut of *A*. *longirostris* in the hydrothermal vent of Okinawa Trough, and that at least two major carbon fixation pathways are present in both gill and gut communities, however, the abundances of these pathways significantly differ between tissues. These evidences support the notion that gill- and gut-associated bacteria contain autotrophs that may serve as important providers, to different extents, of nutrition to the host in hydrothermal systems. Further investigation is required to assess, via approaches such as fluorescence in situ hybridization, the location and amount of the associated bacteria to determine whether the detected sequences represent true epibionts or transient associates of the gill and gut.

## Supporting Information

S1 Fig Venn diagram of the OTUs in the gill and gut samples. Unique and shared OTUs among the ten samples are based on 97% identity. The numbers indicate the numbers of OTUs.
(DOCX)

S1 Table Genera detected in gill and gut libraries of *Alvinocaris longirostris*. +, Detected; -, Not detected.
(DOCX)
