# Supplementary material for: First Comparative Analysis of the Community Structures and Carbon Metabolic Pathways of the Bacteria Associated with Alvinocaris longirostris in a Hydrothermal Vent of Okinawa Trough
Source: PLoS One. 2016 Apr 25;11(4):e0154359. doi: 10.1371/journal.pone.0154359 (PMC4844111; doi:10.1371/journal.pone.0154359)

**Supplementary data**

**Figure S1.** **Venn diagram of the OTUs in the gill and gut samples.** Unique and shared OTUs among the ten samples are based on 97 % identity. The numbers indicate the numbers of OTUs.


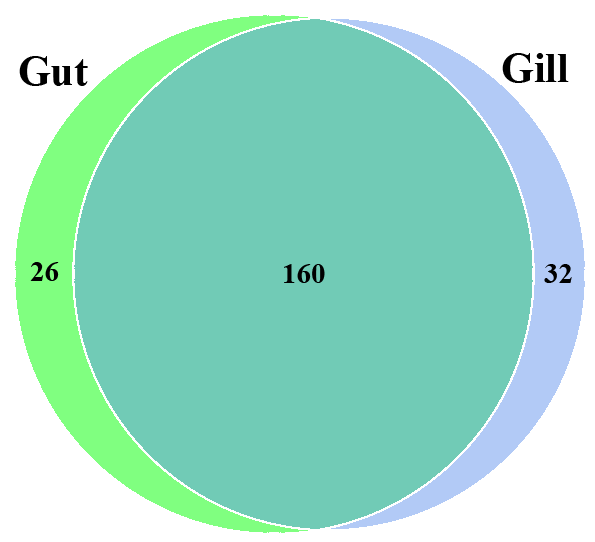

Supplement: S1 Fig — Unique and shared OTUs among the ten samples are based on 97% identity. The numbers indicate the numbers of OTUs. (DOCX) [file pone.0154359.s001.docx]
