# Supplementary material for: First Comparative Analysis of the Community Structures and Carbon Metabolic Pathways of the Bacteria Associated with Alvinocaris longirostris in a Hydrothermal Vent of Okinawa Trough
Source: PLoS One. 2016 Apr 25;11(4):e0154359. doi: 10.1371/journal.pone.0154359 (PMC4844111; doi:10.1371/journal.pone.0154359)
Supplement: S1 Table — +, Detected; -, Not detected. (DOCX) [file pone.0154359.s002.docx]

**Table S1. Genera detected in gill and gut libraries of *Alvinocaris longirostris*.** +, Deteced; -, Not detected.

| \| Genus \| Gill \| Gut \| \| --- \| --- \| --- \| \| *Psychromonas* \| + \| + \| \| *Piscirickettsia* \| + \| + \| \| *Leucothrix* \| + \| + \| \| *Sulfurimonas* \| + \| + \| \| *Sulfurospirillum* \| + \| + \| \| *Pseudomonas* \| + \| + \| \| *Photobacterium* \| + \| + \| \| *Arcobacter* \| + \| + \| \| *Shewanella* \| + \| + \| \| *Loktanella* \| + \| + \| \| *Desulfocapsa* \| + \| + \| \| *Salinispora* \| + \| + \| \| *Gluconobacter* \| + \| + \| \| *Rhodococcus* \| + \| + \| \| *Phaeobacter* \| + \| + \| \| *Acinetobacter* \| + \| + \| \| *Bdellovibrio* \| + \| + \| \| *Methylobacterium* \| + \| + \| \| *Escherichia* \| + \| + \| \| *Sphingomonas* \| + \| + \| \| *Enhydrobacter* \| + \| + \| \| *Thalassobius* \| + \| + \| \| *Pseudoxanthomonas* \| + \| + \| \| *Polaribacter* \| + \| + \| \| *Desulfococcus* \| + \| + \| \| *Sulfuricurvum* \| + \| + \| \| *Brevundimonas* \| + \| + \| \| *Persicirhabdus* \| + \| + \| \| *Bacillus* \| + \| + \| \| *Luteolibacter* \| + \| + \| \| *Lutimonas* \| + \| + \| \| *Acidovorax* \| + \| + \| \| *Rubritalea* \| + \| + \| \| *Rhodobacter* \| - \| + \| \| *Deinococcus* \| + \| + \| \| *Cupriavidus* \| - \| + \| |
| --- | --- | --- | --- | --- | --- | --- | --- | --- | --- | --- | --- | --- | --- | --- | --- | --- | --- | --- | --- | --- | --- | --- | --- | --- | --- | --- | --- | --- | --- | --- | --- | --- | --- | --- | --- | --- | --- | --- | --- | --- | --- | --- | --- | --- | --- | --- | --- | --- | --- | --- | --- | --- | --- | --- | --- | --- | --- | --- | --- | --- | --- | --- | --- | --- | --- | --- | --- | --- | --- | --- | --- | --- | --- | --- | --- | --- | --- | --- | --- | --- | --- | --- | --- | --- | --- | --- | --- | --- | --- | --- | --- | --- | --- | --- | --- | --- | --- | --- | --- | --- | --- | --- | --- | --- | --- | --- | --- | --- | --- | --- | --- |
